# Implementation of Brief Admission by Self-Referral in Child and Adolescent Psychiatry in Sweden: Insights from Implementers and Staff

**DOI:** 10.3390/ijerph21010035

**Published:** 2023-12-26

**Authors:** Björn Axel Johansson, Eva Holmström, Sofie Westling, Sophia Eberhard, Olof Rask

**Affiliations:** 1 Region Skåne, Psychiatry, Habilitation & Aid, Child and Adolescent Psychiatry, Regional Inpatient Care, Emergency Unit, 20502 Malmö, Sweden; eva.l.holmstrom@gmail.com (E.H.); sophia.eberhard@skane.se (S.E.); olof.rask@med.lu.se (O.R.); 2 Department of Clinical Sciences Lund, Division of Child & Adolescent Psychiatry, Lund University, 22100 Lund, Sweden; 3 Department of Clinical Sciences, Malmö, Psychiatry, Lund University, 22100 Lund, Sweden; sofie.westling@med.lu.se; 4 Office for Psychiatry and Habilitation, Psychiatric Clinic Lund, Region Skåne, 22185 Lund, Sweden

**Keywords:** child and adolescent psychiatry, brief admission by self-referral, implementation

## Abstract

Brief admission by self-referral, which allows patients to briefly admit themselves to a psychiatric ward, is a crisis intervention designed to reduce suicide and self-harm. This method was introduced in Sweden for adult patients in 2015, achieving high patient satisfaction and good acceptance among staff. In 2018, the method was adapted and implemented in pediatric psychiatry. The present study comprehensively describes the multifaceted strategies for implementing brief admissions, including planning, education, financing, restructuring, quality management, and policy implementation and reform. It also includes staff’s opinions of the practice of brief admissions for young people. Neither of these topics has been addressed in the existing literature. During the study period (April 2018–April 2021), 63 brief admission contracts were established. The number of new contracts increased exponentially (12.7%) per quarter (*p* < 0.05), and staff satisfaction with both the implementation and its benefits for unstable patients was high. Brief admission by self-referral can be successfully implemented in pediatric psychiatry and appears to be a functional crisis management method for adolescents.

## 1. Introduction

Child and adolescent psychiatric care is generally provided in outpatient settings; however, a small group of patients occasionally requires inpatient care to stabilize life-threatening conditions [1,2]. The first days of emergency admission seem to add the most value, while longer admissions carry risks of symptom escalation, contagion of self-harm, and disconnection from friends, family, and school [1,3,4]. For emotionally unstable, often self-harming, or suicidal patients, the pathway to admission may be perceived as a struggle that is, at times, marked by rejection at some times and involuntary admissions at others. This carries risks of symptom escalation, coercive measures, and a prolonged course of illness [5,6]. Despite the American Psychiatric Association (APA) approving the diagnosis of borderline personality disorder for those under 18 years old, this diagnosis is rarely used for young patients in Swedish clinical practice as symptoms often prove difficult to distinguish from those representing anxiety and affective dysregulation common in adolescence [7,8]. Nonetheless, most patients with a history of severe self-harm fulfill one or more diagnostic criteria for borderline personality disorder, such as emotional instability or unstable relationships with a fear of being abandoned, which are often managed by self-harm and suicidal behavior. Moreover, when left untreated, significant impairments are common [9,10,11,12]. Severe self-harm and suicidal behaviors among adolescents are risk factors for suicide, which, according to the WHO, is the fourth leading cause of death among young people worldwide.

An increasing number of adolescents with severe self-harm, recurrent emergency visits and admissions, and complicated courses, including coercive measures, have been seen in the regional pediatric psychiatric inpatient unit in Malmö in recent decades. A contributing factor to these complicated courses may have been the difficulty in organizing seamless teamwork between inpatient and outpatient operations. Coordinated treatment plans to prevent recurring crises are often lacking, partly because of the cultural differences between 24/7 and outpatient care [13,14]. Alternatives to admissions were found to be insufficient, and admissions with longer duration than anticipated were frequent [11,13]. In 2015, the treatment gap concerning the most unstable and self-harming patients was addressed, and a decision was made to develop new approaches to improve support and treatment for this patient group.

In the Netherlands, an initiative allowing patients to refer themselves to inpatient treatment was introduced in psychiatric care in the 1990s [15], aiming to support emotional regulation and increase self-control and autonomy, thereby reducing suicidal behavior and severe self-harm. The intervention was called “bed-on-prescription” in Dutch, which was translated to “brief admission by self-referral” in later research. It consisted of admissions that were limited in duration and frequency as part of a negotiated care plan and were offered to both adults and adolescents. In 2015, the method was adapted and implemented in Swedish adult mental health services by Flyckt et al. [16,17,18] at Karolinska Institute in Stockholm and Westling et al. [19,20] at Lund University; the method demonstrated both high levels of patient satisfaction among individuals using the intervention and good acceptance among staff [17,18].

These promising results piqued the interest of clinicians at the regional department of child and adolescent psychiatry in Malmö, who then inquired whether the method could also be beneficial to Swedish adolescents. In 2016, it was decided that the method would be adapted for adolescents, allowing for brief admissions to be implemented at a unit that serves 300,000 children and adolescents aged 0–17 years in Skåne. In a recent study, Johansson et al. (2023) showed that brief admissions as a crisis management method for adolescents were associated with a reduced need for emergency psychiatric care, i.e., significant reductions in emergency visits, emergency admissions, and inpatient days, including brief admissions [7].

The primary objective of the present study was to describe the implementation process for brief admission at the Child and Adolescent Psychiatric Emergency Unit in Malmö, Sweden, to inspire other units that wish to enhance their services with this intervention. The secondary aims were to describe the characteristics of the adapted brief admission concept for adolescents and assess how the intervention was perceived by the staff.

## 2. Materials and Methods

### 2.1. Ethical Approval

This study was part of a quality improvement project that posed no risk to the participants. The study protocol was approved by the Swedish Ethical Review Authority (2021-03640).

### 2.2. Implementation Processes

The multifaceted implementation strategies included planning, education, financing, restructuring, quality management, and policy development and reform [21]. These strategies were the building blocks of the implementation process [22] (Table 1).

### 2.3. Planning

#### 2.3.1. Initiation

To address the treatment gap regarding severely self-harming adolescents with repeated hospitalizations, an implementation team of researchers, clinicians, and clinical management personnel, all with special interest in this group of patients, was formed in 2016. They were tasked with assessing the possibility of adapting the Swedish version of brief admission to suit adolescents.

#### 2.3.2. Executive Boards

To implement an intervention supporting the autonomy of a suicidal patient, partly implicating the withdrawal of physician control, the executive board was required to be involved. For brief admissions, nurse aides became the primary contacts who were involved in admission and discharge. To do so, the nurses needed profound and outspoken support from the managers, as well as support from a senior psychiatrist on demand. The manual for implementing brief admissions states that the executive board must approve and support the implementation of brief admissions [19]. Thus, the executive boards of the clinic and senior psychiatrists were involved throughout the implementation process, actively participating in its planning, structure, and evaluation.

#### 2.3.3. Collaboration among Units with Brief Admission Experience

While brief admission was not available for children and adolescents in Sweden, it was an established crisis intervention in Utrecht, the Netherlands, to support both adults and adolescents who were struggling with emotional instability and self-harm. In February 2017, a brief admission implementation team was invited to the University Hospital in Utrecht to learn about the intervention and was given the opportunity to meet patients with access to brief admissions. The visit provided a comprehensive understanding of the method’s uniqueness, including its potential benefits and pitfalls, which were clearly expressed by the patients and their caregivers.

#### 2.3.4. Feasibility Study—Could This Also Work for Adolescents in Sweden?

After the study visit, we decided to start adapting the adult Swedish version of brief admission to suit adolescents in Region Skåne, Sweden. Six patients were included in a pilot study and gained access to brief admissions, using a preliminary protocol inspired both by the adult manual for implementation in Lund [19] and by the adult manual with adaptations used for patients in Utrecht (M Helleman, lecture, 13 February 2017). The included patients were all well-known at the emergency unit in Malmö and presented with complex psychiatric symptoms, including self-harm; at least 3/9 diagnostic criteria for borderline personality disorder, often with repeated emergency visits; and a medical history with more than one inpatient admission. The evaluation after 12 weeks demonstrated that the preliminary protocol was successful and that a clear manual on staff procedures for brief admissions was required. The overall impression was that the brief admission concept is feasible for implementation in child and adolescent psychiatry in Sweden.

### 2.4. Education

#### 2.4.1. DBT Training

In both Utrecht and Malmö, dialectic behavioral therapy (DBT), along with its approach of validation and strategies to reduce self-harm, was the first-line treatment for patients who qualified for brief admissions [23,24]. The decision was also made to strengthen competency in teaching DBT skills among staff at the Malmö inpatient unit. A brief admission team was established, consisting of a senior consultant (author EH) and two mental health nurses’ aides, all of whom took part in a DBT training course from 2017 to 2018 that was taught by Professor Alan Fruzzetti from Harvard Medical School and his Swedish DBT colleagues. After the training, the brief admissions team met regularly for internal supervision to strengthen their competence in validation and emotional skills. Later, the brief admission team developed a structured skills training program that was adapted to be used three times a week with patients who were undergoing either brief or emergency admissions.

#### 2.4.2. Brief Admission Team

The brief admission team attended a 3-day “training for trainers” course in brief admission, including the 1-day, manual-based, standardized training in brief admission that was offered to the staff of adult units that were implementing brief admissions. The training program was developed and delivered by an adult brief admission research team at Lund [19]. The brief admission team updated the methodology for staff procedures in the preliminary protocol that was based on the manual for adults [19] and initiated a 1-day recurrent training program for all staff at the emergency unit.

#### 2.4.3. Brief Admission Training of the Staff at the Emergency Unit

The brief admission team became responsible for structured and continuous internal education, evaluation, and support to reduce the risk of methodological drift. They were guided by experiences from both the department in Utrecht and the adult psychiatric research team in Sweden, which emphasized the importance of continuous follow-up during implementation. In accordance with implementation in adult psychiatry, shorter training was given to staff who were not directly involved in providing care to the brief admission patients, such as administrative staff, to allow for questions and dialogue; booster sessions in small groups have since been provided twice a year to ensure that new staff are introduced to the method and to update senior physicians [19,20].

#### 2.4.4. Staff at the Outpatient Units

During the implementation, the brief admission team collaborated closely with the region’s 18 psychiatric outpatient units and some residential treatment facilities to continuously update their staff on brief admission. The team members presented the central themes of the intervention and the practical aspects of the method. These meetings provided the staff with the opportunity to ask questions and discuss the methods.

### 2.5. Finance

This brief admission implementation was mainly funded by the Region Skåne Health Care Authority, which is aligned with the identified treatment gap and expressed the ambition to improve the available treatments for severely self-harming patients with repeated hospitalizations. Additionally, external funding was granted by the Lindhaga Foundation to cover the costs of the study visit to The Netherlands.

### 2.6. Restructuring

#### Changes in the Physical Structure of the Ward and the Record System

In the emergency unit, two rooms were designated for patients with brief admission contracts. On the rare occasions when both of those rooms were occupied when a patient contacted the emergency unit, a discussion was initiated with the patient about whether there were sufficient coping strategies in place to deal with anxiety at home until a new contract could be made the following day. If this was not possible, the patient was encouraged to undergo regular emergency assessments. In addition to changes in the structure of the emergency unit, clinical documentation of all patients was performed by a dedicated senior physician.

### 2.7. Quality Management

Quality management was guided by an implementation manual [21,22] and the experiences of the adult brief admission team. The brief admission team had regular meetings in which each patient with the potential to be included was discussed. Once included, collegial supervision and support were established, preferably by colleagues from the adult team in Lund. At discharge, the patients were asked to assess three statements concerning the method on a Likert scale [7]. The figures and numbers of the included patients were closely monitored by clinical management. An important part of the implementation process included scheduled weekly sessions, allowing brief admissions team members to reflect on both the challenges, such as different forms of resistance to new procedures, and on what worked well. These meetings were also important for reducing the risk of drift in the method. Overall feedback on progress was given to staff both at the unit and in open care units every 6 months to empower the implementation process. One year after the implementation ended, employees were asked to anonymously rate their level of agreement with three statements (A–C) about their experiences with the brief admissions implementation process. It is my understanding that A The Brief Admission implementation process went well, B Brief Admission today is an accepted method among staff, and C Unstable patients benefit from Brief Admission. The three statements were rated on a Likert scale ranging from 0 (strongly disagree) to 3 (strongly agree). The maximum composite score was nine points.

### 2.8. Policy Development and Reform

Owing to staff turnover and to maintain focus, the brief admission team members regularly visited different outpatient units after implementation to discuss brief admissions and encourage the use of the method in the region.

### 2.9. Characteristics of the Brief Admission Concept for Adolescents

#### 2.9.1. Eligibility Criteria

For an adolescent (aged 13–17 years) to be eligible for a brief admission contract, either a history of previous emergency unit visits or a previous inpatient admission in the previous 6 months was required, together with a complex psychiatric condition with features of emotional instability and recurrent suicidality that corresponded to at least three of the nine criteria for borderline personality disorder. Additionally, patients needed to understand the implications of the contract. They were also required to have ongoing outpatient treatment contact [20]. For patients, it was decided that this should be limited to structured treatment programs, such as DBT or cognitive behavior therapy (CBT), with a focus on emotional regulation skills and anxiety management strategies.

#### 2.9.2. Exclusion Criteria

Patients with intellectual disabilities or psychotic syndromes and those who did not understand Swedish were excluded, as were those cared for in state institutions for legal reasons. Additionally, patients temporarily placed in emergency homes were excluded due to uncertainty about their future [7]. Patients with unstable housing or who did not have regular outpatient care were also excluded.

#### 2.9.3. Patient Recruitment

The most significant differences between brief admissions and emergency admissions in pediatric psychiatry are summarized in Table 2. Patients eligible for brief admission were most often identified during the morning rounds at the emergency unit but were also identified in outpatient units. When outpatient care was insufficient, for example, in the context of recurrent difficult-to-control crisis reactions and self-harm, the outpatient therapist consulted with the brief admission team to discuss the possibility of a brief admission arrangement. A brief admissions team assessed the requests. If a brief admission seemed suitable, feedback was provided to the therapist at the outpatient unit for further discussion with the patient and their caregivers. If all agreed to the enrollment, the next step was to negotiate an individualized brief admission contract and book an appointment for contract signing (Appendix A). This occurred in the patient’s outpatient unit. Occasionally, patients themselves were inspired by their fellow patients and took the initiative to apply for a brief admission contract, either during inpatient treatment or when visiting open care. The applications were handled in accordance with the standard brief admission procedures described above.

#### 2.9.4. Introduction to the Concept

When a decision was made to enroll an adolescent, a member of the brief admission team visited the outpatient unit to introduce the framework of the brief admission contract to the patient, their legal guardians, and a therapist in the outpatient unit. This concept is based on the practice of the brief admissions of adults [19]. The differences are summarized in Table 3.

#### 2.9.5. Negotiating and Signing the Contract

Once the patient was informed about and understood the framework of the brief admission contract, the next step was to negotiate its content. Negotiation is standardized [19] and requires that the adolescent can focus for at least 45 min; furthermore, it is imperative that the scheduled appointment takes place in a safe and calm room, which provides a sense of importance and respect. During the negotiations, electronic devices were switched off to make it easier to focus on the task. Many things were discussed, such as overarching six-month goals for passing school, stopping self-harm, and generally achieving less problematic behavior. Early signs of deterioration, such as increased irritability, impaired sleep, or disturbed eating habits, were highlighted, as well as when, using personal crisis intervention strategies, brief admission should be activated. The preferred approach from staff during a brief admission was addressed, such as, for example, if the patient preferred to be left alone or encouraged to socialize, to be woken up or not in the morning, or how they wanted to be reminded of their anxiety management strategies in the case of acute anxiety. During the negotiation, five themes were always emphasized: following the ward rules and routines, no sharp objects, no alcohol or drugs, asking for and receiving help when needed, and abstaining from self-harm or harming anyone else [19]. During admission, the content of the individualized contract guided the staff in the inpatient unit. A copy of the contract was always provided to the patient and their legal guardian, and another copy was stored in a secure location in the nurse’s office at the inpatient unit, which was easily accessible if the patient requested a brief admission. In addition, a copy was added to the patient’s medical records.

#### 2.9.6. Coming to the Unit for a Brief Admission

Routines for initiating a brief admission follow those for adults in many ways [19]. Adolescents should always contact the emergency department via phone and announce their arrival at the hospital. A brief admission patient is usually in crisis when self-admitting, and it can be challenging for them to make the call; therefore, the staff should always be affirmative and welcome. Brief admissions should not be questioned. Adjacent to the phone call, the staff prepared themselves by reading the brief admission contract, and when the patient arrived, an enrollment conversation was performed with the staff without delay. Patients never sat in the waiting room with other patients for an extended period. Brief admission patients were greeted with warmth and respect, such as, for example, by saying, “It’s so good that you chose to come.” It is important that the staff take a truly empathic approach so that the patient’s experience of the situation can be the focus [25,26]. The patient’s questions were answered directly, and they were not criticized; validating their anxiety and pain is important. Unlike brief admissions for adults, a security check was performed during brief admissions for adolescents, where the patient is asked to show the contents of their pockets and belongings. The patients were asked if they had brought drugs or sharp objects. Cigarettes and lighters were secured in a personal locker. After the security check, the staff sat down with the patient and reviewed the contract, focusing on safety, anxiety management strategies, and admission goals. The date and time of discharge were determined, and the maximum length of stay was three days. The legal guardians and a senior consultant at the emergency unit were informed about the time of discharge.

#### 2.9.7. During the Brief Admission

Ward routines for adolescents were like those for adults in many ways [19]. The most significant difference was that adolescents, in their contract, could state if they wanted to bring a parent with them during a brief admission. After admission, a treatment plan guided by the patient’s contract was written. During admission, the patients were not examined by the unit’s doctors or psychologists. Night leaves were not granted. Any medication adjustments, therapeutic sessions, or changes in the contract occurred in outpatient units. Each day, the patient had the opportunity for two 20-min sessions with staff, focusing on the “here and now” situation, e.g., “Why escalation?” and “Did you use your emergency plan?”. During admission, it is desirable that patients, if possible, take responsibility for their outpatient treatment and education despite their crises; for example, they could take local transportation to and from their outpatient units and schools. Some of these sessions could be conducted digitally. Patients could, if they wished to, eat in their separate rooms; sometimes, they were advised not to interact with other patients. An important difference between adolescents and adults is that a unit nurse handles a patient’s medication and can support staff in not leaving the patient alone when there is an urge to self-harm. However, it is also important to develop alternative strategies to combat anxiety. Collaboration with outpatient units is emphasized.

#### 2.9.8. Discharge Procedure

Parents were involved in the discharge process. During the discharge procedure, which is led by a brief admission team member, the course of the stay was summarized, and the treatment plan was evaluated, asking questions like “What went well?” and “Which parts could be improved?”. A brief questionnaire regarding patients’ experiences was completed [19]. Unlike adults, a suicide risk evaluation was performed with adolescents by a brief admission team member, if required, with support from a brief admission team physician, who always made a final note in the patient’s medical record. When a patient was affected by any substance during a brief admission or otherwise violated the contract (e.g., threatening staff or a fellow patient), they were forcefully discharged. It is considered important not to diminish the seriousness of violations (Table 2) [19].

#### 2.9.9. Termination of Contract

Every contract was reevaluated biannually by the patient, their open-care therapist, and a brief admission team member, which was more often compared with adult contracts [19]. Some brief admission patients opted out because they were unable to fulfill the contract requirements. If the patient improved and was discharged from open care treatment, the brief admission contract was automatically terminated; otherwise, it usually ended with an evaluation when the patient turned 18 years old. If the patient was to be referred to adult psychiatry for continued care after their 18th birthday, the final report mentioned that the patient had a brief admission contract and included the results of this intervention. Colleagues in adult psychiatry then assessed whether the individual should receive a contract within the framework of their program. The differences between the brief admissions in adult and pediatric psychiatry are summarized in Table 3.

## 3. Results

### 3.1. Early Clinical Experience and Outcome of Brief Admissions for Adolescents

#### 3.1.1. Characteristics of Patients with Brief Admissions

During the implementation period, between April 2018 and April 2021, 928 patients were admitted to the emergency unit; of these, 60 were considered ineligible due to the reasons described in Section 2.9, and 801 patients did not meet the inclusion criteria, leaving 67 eligible participants [7]. Four patients declined to sign a brief admissions contract, and 63 patients were subsequently included. A total of 10 different diagnoses were identified, with the four most common being major depressive disorder (38%), anxiety disorder (19%), severe stress (14%), and attention-deficit hyperactivity disorder (8%) [7] (Table 4).

Of the 67 patients who were eligible for inclusion during the implementation period, four were omitted. One declined a contract because the requirements were perceived as too difficult. Another participant had to cancel the contract soon after inclusion because their parents could not accept that the patient could be left unsupervised during future brief admissions. A third patient was not included because the parents could not accept the gender-integrated ward. The fourth patient moved to another healthcare region [7].

**Figure 1:**
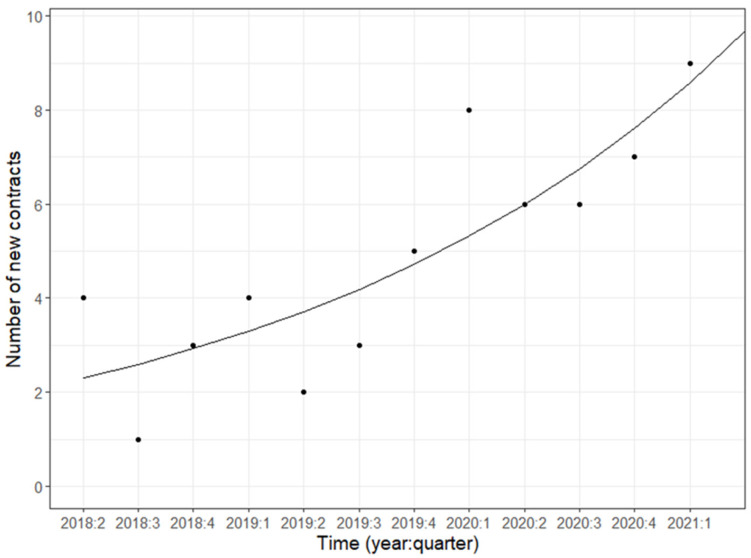
The number of newly signed contracts per quarter (except the last), plus the trend line for the expected number of new contracts per quarter: y = 2.04 × 1.127^x^, where x = number of quarters after 2018:1, i.e., 2018:2 is x = 1. This means that the number of new contracts increased (*p* = 0.0028) by 12.7% per quarter on average. Calculated using Poisson regression.

Thus, 63 brief admission contracts were established between April 2018 and April 2021. The time between the patients’ first visits to the emergency department and signing the contracts was a median of 9.6 (4.3–23.7) months [7]. On average, the number of new contracts increased exponentially (by 12.7%) per quarter (calculated using Poisson regression; *p* = 0.0028) (Figure 1).

#### 3.1.2. Staff Experience Measures

A total of 80 co-workers, all of whom worked with this patient category in either round-the-clock care or outpatient units for at least 6 months during 2019–2020, were identified from six professional categories (executives, physicians, psychologists, counselors, nurses, and staff). A total of 66 of these professionals had available contact information (including current e-mail addresses) and were contacted by the administrative staff, who then collected and compiled the data at the group level. Employees who did not respond to the first email received reminders on a maximum of two occasions.

Of the 66 employees, 41 (62%) scored the three statements with an average total score of 8.2 points, indicating very high satisfaction with A the brief admission implementation (mean 2.55 points; range 2–3), B present acceptance of the method (mean 2.83 points; range 2–3), and C its benefits for unstable patients (mean 2.95 points; range 2–3) as assessed by the staff. Among the 41 respondents, three were executives, 10 were physicians, eight were psychologists, two were counselors, six were nurses, and 12 were nurses’ aides. No group differences were found with respect to vocations. Among the 41 respondents, 17 worked in the 24/7 service and 24 in the outpatient organizations. No differences were observed between the groups.

## 4. Discussion

To address the treatment gap regarding admissions for severely self-harming adolescent patients, an initiative was undertaken to assess the possibility of exploring and implementing the brief admission concept for these patients. In our experience, as outlined in this study, adaptations to the brief admission model for adult psychiatry can be applied successfully to crisis management for adolescent patients.

Previous studies on both adult and adolescent patients have demonstrated a reduced need for inpatient care with brief admissions [7,27,28,29]. These results have not yet been confirmed [20,30,31]. The present study reports on the implementation process of brief admissions to the Child and Adolescent Psychiatric Emergency Unit in Malmö, Sweden. With brief admissions, the patients seemed to experience psychiatric treatment with clear elements of self-determination and participation [25]. The patients’ enhanced sense of autonomy is supported by the lack of restrictions and a “welcoming, non-authoritarian attitude” from the staff [13]. The present study additionally reports that the intervention was well appreciated by the staff involved in pediatric psychiatric care. Brief admissions may bridge the gap between inpatient and outpatient care [13]. In this paper, we summarize the cornerstones of our work. To our knowledge, no previous study guidelines for the implementation of brief admissions in pediatric psychiatry have been published, nor has there been a study on staff reception of the method.

### 4.1. Main Findings

The implementation process was successful, and the staff experience was positive. In a previous study by Eckerström et al. [18], brief admissions for adults demonstrated good acceptance among eight informants representing round-the-clock care [18]. In the present study, 41 round-the-clock and outpatient care employees provided information, all of whom were satisfied with the method. The number of signed contracts increased exponentially during the implementation period, also indicating that employees and colleagues had faith in the method (Figure 1).

The implementation of a new model of care is an ongoing process, usually lasting between 2 and 4 years [21,22]. The process usually begins with a needs inventory and ends with maintenance of the method. One contributing factor to our successful implementation may be that we were guided by strategies and recommendations (Table 1).

Most of the implementation strategies used were planned based on the experiences from implementing brief admissions in the Netherlands as well as in adult psychiatry. Some additional strategies were added as a result of the feasibility study that highlighted the need for a structured, manual-based procedure. Our strategies, e.g., the study visit and pilot study, essentially recalled the points Powell et al. [21,22] present on why it felt natural to incorporate these concepts and the recommended terminology into our work.

Among teens, impulsivity, dissociation, and affective dysregulation, representing anxiety and affective disorders, can be difficult to distinguish from the early presentation of a borderline personality disorder [12]. In our emergency unit, all patients are welcome, but only the most affected were included in the study, explaining the gap between eligible and included patients. Eight hundred one of the 868 potentially eligible patients lacked a complex psychiatric picture with instability and recurrent suicidal behavior corresponding to at least 3/9 criteria on borderline personality disorder and/or were younger than 13 years and/or visited the emergency unit for the first time.

Initially, during the implementation process, several critical concerns were raised: could the patients, with their prominent vulnerability, independently take responsibility for a brief admission contract? Another concern was whether it would be feasible to overcome skepticism among the employees regarding a new methodology. A third obstacle was the bother about our ability to maintain methodological fidelity during the several-year implementation process. However, we trusted the method, and patients showed that they, regardless of their vulnerability, were able to handle the increased responsibility; we provided recurring training efforts for staff, and we ensured that representatives from the brief admission team were present in the whole process. We learned to emphasize the importance of repeated training at different levels in the organization. We also learned that the value of dedicated brief admission team members cannot be underestimated. Integrated and ongoing supervision to maintain method fidelity was central. Patient evaluations and contributing with constructive feedback also increased motivation for ongoing implementation.

### 4.2. Achievement Factors

Achievement factors were outlined guided by previous literature and our experiences of what contributed to implementation success. First, an important achievement factor was our use of a model that was recently and successfully implemented in a Swedish adult psychiatric setting for inspiration, which meant that we could, after adaptation, implement the same model for adolescents [20]. The colleagues in the adult brief admissions team in Lund were generous in sharing their knowledge and experiences. Second, the operations manager was confident about the project, with an attitude that spread throughout the organization and helped us obtain a supportive organization [22]. Third, the brief admissions team had dedicated members. Their interest in the task contributed to the fidelity of the method. Fourth, standardized education for the entire organization was another key factor [19]. Both brief admissions and DBT teaching were guided by the respective national and international authorities. The brief admission team educated the emergency unit staff and the staff in the open care units. The training efforts contributed to the staff’s curiosity about brief admission [22]. Early in the process, the staff developed a learning attitude, common vision, and belief in change. Thus, implementation soon became an interactive process with the staff [22]. Fifth, the unit already had experience in implementation work, which paved the way for brief admissions in terms of content education. Sixth, regular self-supervision for 1.5 h a week with the clinically present brief admission group was also considered an achievement factor. Finally, positive feedback from patients and caregivers provided encouragement throughout the process.

### 4.3. Hypothetical Earnings, Strengths, and Limitations

Brief admissions for adolescents with severe emotional dysregulation hold promise as a crisis management method that could reduce the risk of hospitalizations and coercive measures and increase treatment care quality and availability [7]. All the staff in the emergency unit participated in the implementation process; that is, this study describes the implementation process in a clinical setting. The implementation work is important because it is based on real-life situations. A limitation of the present study is the lack of a control group; however, the design must be considered the best possible, and suitable control groups are often difficult to mobilize in implementation research [32]. The majority of participants in the study were girls, limiting generalizability for boys. Although the sex gap in the present study is prominent, it is representative of treatment studies on other populations targeting adolescents with emotion dysregulation, self-harm, and suicidality among adolescents [23,33].

### 4.4. Future Implications

It is hoped that this study will inspire implementation in other units and further research on brief admissions in child and adolescent psychiatry. Other patient categories, such as patients with psychotic syndromes [29] and eating disorders [28], who have shown promising results in adult psychiatry, might also be included using special contracts, as well as non-Swedish patients.

## 5. Conclusions

Brief admissions can be successfully implemented in pediatric psychiatry and appear to be a functioning crisis management method for adolescents with severe emotional dysregulation.

## Figures and Tables

**Table 1:** Implementation strategies used.

Implementation Strategy	Application
Planning	
Build a coalition	The goal was to organizing an implementation team with providers, managers, and stakeholders at different levels for children and adolescents in the region.
Conduct local consensus discussions	The idea to implement the model came from a significant clinical problem, and the implementation team discussed whether brief admissions could be appropriate for the problem.
Involve executive boards	The executive board of the clinic as well as the senior psychiatrists were involved throughout the implementation process, actively participating in planning, structure, and evaluation.
Visit other sites	The team organized a study visit to the Netherlands where a model for brief admission was in use in an adult and adolescent psychiatric setting.
Assess for readiness and identify barriers and facilitators	The implementation team suggested forming a brief admission team with the goal to adapt and implement brief admission for children and adolescents in the region.
Develop academic partnerships	The implementation process was completed in collaboration with researchers from the Department of Child and Adolescent psychiatry and the Department of Adult Psychiatry at Lund University.
Stage implementations scale up	Using a preliminary protocol, six patients were included in a pilot study and gained access to brief admission.
Education	
Create a learning collaborative	A brief admission team was formed that included a senior psychiatrist and two nurses’ aides.
Use train-the-trainer strategies	The brief admission team members attended the training for trainers in the adult brief admission model.
Develop educational materials	The brief admission team, guided by the adult team, adapted the education manual to be suitable for adolescents.
Work with educational institutions	The brief admission team members took part in a DBT training course held by an international authority and his Swedish DBT-colleagues.
Conduct ongoing training	The team completed initial comprehensive training of all staff working in the ward, then repeated trainings to introduce new staff members and give updates to senior staff.
Conduct educational meetings	Brief lectures were held for providers in adjacent units, administrators, and other organizational stakeholders.
Provide ongoing consultation	The adult brief admission team held continuous consultations to facilitate the implementation process.
Finance	
Access new funding	External funding was sought for a study trip to the Netherlands.
Restructure	
Change physical standards and equipment	Two rooms that were dedicated for patients participating in brief admissions were prepared.
Change record systems	Clinical documentation for all patients was made by a dedicated senior physician.
Quality management	
Audit and provide feedback	Forms that were adapted for adolescents were prepared to investigate satisfaction with the model.
Capture and share local knowledge	The unit had experiences from previous implementation processes with other models.
Organize clinician implementation team meetings	The brief admission team had recurrent meetings with the implementation team to reflect on the implementation effort, share lessons learned, and support each other’s learning
Provide clinical supervision	A clinician supervised the implementation. The brief admission team members were trained to ensure that they had the skills needed to supervise staff
Policy development and reform	
Create or change credentialing and standards	The team created an operation that encouraged using the method. The work to make this change continues through training requirements that encourage continued and increased use of the method.

**Table 2:** Differences between brief admissions and emergency admissions in child and adolescent psychiatry.

	Brief Admission by Self-Referral	Emergency Admission
Before admission		
Information	Information about brief admission	Information about our 24/7 emergency service
Contract negotiation	Individual parts are reviewed: e.g., overreaching six-month goals, early signs of deterioration, preferred approach from staff, and stress-reducing activities	-
Contract	The contract is written, and signed by the patient, a parent, the open care therapist, and a brief admission team member	-
Approach from staff	Affirmative and welcoming, emphasizing validation	Validation, respect
Admission	Patient decision according to contract	Admission after physician’s decision
During admission		
Security check	The patient shows what is in their bags and pockets. Cigarettes and lighters are taken and secured	The patient shows what is in their bags and pockets. Cigarettes and lighters are taken and secured
Contract reading and treatment plan	The individual contract is read together with a nurse and a treatment plan is written	A treatment plan is written together with patient and parents
Treatment length	1–3 days, maximum three times/month	An average of seven days
Accompany parents	If written in the contract	A parent is admitted with the patient
Meds—administration	By unit nurse	By unit nurse
Meds—adjustments	Adjustments by the open-care physician	Possible, if unit physician considers it justified
Daily staff support	20 min two times per day	Irregularly depending on need
Meals	Can eat in privacy	Shared meals
Evaluation by psychologist	-	Evaluation usually every other day
Evaluation by physician	-	Evaluation usually every other day, also together with a parent
Temporary leaves	Yes, according to contract. If the patient can visit their therapist, school, or leisure activity despite the impending crisis, this is encouraged	Possible after agreement with the unit’s physician
Overnight leaves	No	Possible after agreement with parents and the unit’s physician
Premature discharge	If the patient breaks the contract by self-harming, threatening others, or acting violently	If the patient objects or sabotage offered treatment and not can be converted without coercive care. Symptom escalation often results in an extended treatment period.
Discharge procedure	Discharge interview with a brief admission team member. Parents are informed. A brief questionnaire regarding the admission is completed	Discharge interview with the unit physician, staff, and parent.
Coercive care	-	Can be applied if the patient suffers from a serious mental disorder, e.g., mania, cannot be cared for in any other way than within 24-h psychiatric care, and opposes such intervention.
Counseling for accompanying parents	-	Sometimes the parents ask for counseling, while staff take the initiative at other times. Social services are sometimes contacted about staff concerns
Cooperation	Outpatient care unit	Outpatient care unit, sometimes school, social authorities
Supervision	-	Different degrees of supervision depending on the illness, the patient’s maturity, and their ability to take personal responsibility
Hospital school	-	Sometimes, during prolonged admissions
Unit activities	Voluntary	Encouraged to participate

**Table 3:** Differences between brief admission for adolescents and adults.

	Brief Admission for Adolescents	Brief Admission for Adults
Negotiation	Parents participate	Next of kin do not participate
Security check on admission	Bags checked	Bags are not checked
Parents/next-to-kin	Possibility to admit a parent	Next of kin are not admitted
Medication	Provided and handled by the ward nurse	Brought and handled by the patient
Possibility of supervision	Unit nurse can support staff in not leaving the patient alone when there is an urge to self-harm	No constant supervision
Discharge	Parents involved	No approval needed when the adult wants to be discharged
Contract re-evaluation	Biannually	Annually

**Table 4:** Demographic data, including ICD-10 main diagnosis, in 63 patients included in brief admission concept at the Child and Adolescent Psychiatric Emergency Unit, Malmö, Sweden, April 2018–April 2021.

Demographics	*n*	%
Girls	60	95%
Boys	3	5%
Median length (months) of the pre-brief admission period	9.6 (4.3–23.7)	
Mean age (years) when signing the brief admission contract	14.8 ± 1.7	
F10, Alcohol abuse	1	2%
F31, Bipolar disorder	1	2%
F32, Major depressive disorder	24	38%
F41, Other anxiety disorders	12	19%
F42, Obsessive-compulsive disorder	2	3%
F43, Reaction on severe stress	9	14%
F50, Eating disorders	4	6%
F84, Pervasive development disorders	3	5%
F90, Attention Deficit Hyperactivity Disorder	5	8%
F93, Emotional disorders (Childhood onset)	2	3%

## Data Availability

The data presented in this study are available on request from the corresponding author.

## Appendix A

Contract for brief admission by self-referral at the Department of Child & Adolescent Psychiatry in Malmö, Sweden.

The main goal of brief admission by self-referral is that I can admit myself when there is a risk that I will injure myself or have thoughts of taking my life. With help of my brief admission contract, I can take control of my care, myself, and my situation.

My goals

…

Which are my early signs before calling for a brief admission?

…

How do I get admitted?

I can call the emergency unit.

I can call daily between 8 AM and 8 PM.

If there are no brief admission beds, I can talk to the staff about available alternatives, e.g., the opportunity to call the following day or visit the emergency room.

How does a brief admission stay proceed?

The staff will admit and discharge me.

Together with staff, my specific goals for the admission are determined.

A treatment plan for the stay is written.

I can be admitted for a maximum of three days per admission, an all-out of three times per month.

I may book 20 min long sessions with staff twice a day.

I can participate in the department’s activities.

During the admission, I am responsible for my planned treatment visits in outpatient care.

I get my medication according to my medicine list from a unit nurse.

If possible, I can maintain my scheduled outdoor activities.

During the brief admission stay, I do not see a doctor or a psychologist.

I have access to the same care, e.g., the opportunity to seek emergency consultations that I would have had if I had not used the brief admission contract.

Before discharge, I evaluate the brief admission stay together with staff.

The brief admission contract is assessed and revised every six months.

What can I do during my stay to feel better?

…

How can the staff support me to improve my health and achieve my goals?

…

Is there anything else that is especially important for me during the stay?

…

Is there anything special I need to arrange during my stay?

…

My promises during the brief admission stay are as follows:

I will not bring any potentially dangerous items nor perform violent behavior.

I will ask for and receive help when needed.

I will not hurt myself.

I will not expose others in the unit to risks.

I will not consume alcohol or illegal substances.

I will follow the unit rules.

I have read and understood the content of the contract and will follow it.
Signatures
